# Development of a *Galleria mellonella* Infection Model to Evaluate the Efficacy of Antibiotic-Loaded Polymethyl Methacrylate (PMMA) Bone Cement

**DOI:** 10.3390/antibiotics13080692

**Published:** 2024-07-25

**Authors:** You Zhao, Gopala Krishna Mannala, Raphaëlle Youf, Markus Rupp, Volker Alt, Martijn Riool

**Affiliations:** Department of Trauma Surgery, University Hospital Regensburg, Franz-Josef-Strauß-Allee 11, 93053 Regensburg, Germany; you.zhao@klinik.uni-regensburg.de (Y.Z.); gopala-krishna.mannala@klinik.uni-regensburg.de (G.K.M.); raphaelle.youf@klinik.uni-regensburg.de (R.Y.); markus.rupp@klinik.uni-regensburg.de (M.R.); volker.alt@klinik.uni-regensburg.de (V.A.)

**Keywords:** antibiotic-loaded bone cement, *Galleria mellonella*, prosthetic joint infection, *Staphylococcus aureus*, biofilm, in vivo

## Abstract

Prosthetic joint infections (PJIs) can have disastrous consequences for patient health, including removal of the device, and placement of cemented implants is often required during surgery to eradicate PJIs. In translational research, in vivo models are widely used to assess the biocompatibility and antimicrobial efficacy of antimicrobial coatings and compounds. Here, we aim to utilize *Galleria mellonella* implant infection models to assess the antimicrobial activity of antibiotic-loaded bone cement (ALBC) implants. Therefore, we used commercially available bone cement loaded with either gentamicin alone (PALACOS R+G) or with a combination of gentamicin and vancomycin (COPAL G+V), compared to bone cement without antibiotics (PALACOS R). Firstly, the in vitro antimicrobial activity of ALBC was determined against *Staphylococcus aureus*. Next, the efficacy of ALBC implants was analyzed in both the *G. mellonella* hematogenous and early-stage biofilm implant infection model, by monitoring the survival of larvae over time. After 24 h, the number of bacteria on the implant surface and in the tissue was determined. Larvae receiving dual-loaded COPAL G+V implants showed higher survival rates compared to implants loaded with only gentamicin (PALACOS R+G) and the control implants without antibiotics (PALACOS R). In conclusion, *G. mellonella* larvae infection models with antibiotic-loaded bone cements are an excellent option to study (novel) antimicrobial approaches.

## 1. Introduction

Orthopedic device-related infections (ODRIs) are infections associated with implanted medical devices used in orthopedic surgeries, such as joint replacements (i.e., prosthetic joint infections; PJIs), fracture fixation devices (i.e., fracture-related infections; FRIs), and spinal implants. These infections represent a significant clinical challenge, leading to high treatment failure rates and substantial patient and socioeconomic burdens. These infections can lead to prolonged hospital stays, multiple surgeries, and in severe cases, the removal of the implant. Thus, effective preventive and therapeutic strategies are critical in orthopedic and trauma surgery [1,2].

PJIs are severe complications following joint replacement surgeries, characterized by bacteria adhering to implant surfaces and forming protective biofilms that complicate treatment. These infections occur in about 1–2% of cases, with rising incidence due to the increasing number of surgeries because of the global trend of population aging. Common pathogens found in PJIs include *Staphylococcus aureus*, coagulase-negative staphylococci, and Gram-negative bacteria. The complexity of PJIs arises from biofilm formation on the implant surface and within bone tissue, which reduces the effectiveness of the host immune system and antimicrobial treatments [3]. The rise in antimicrobial resistance (AMR) has increased interest in combination-based therapies and the development of advanced drug delivery technologies [4,5,6]. Antibiotic-eluting technologies have become widely utilized in PJI prevention and treatment, as they deliver high, sustained local antibiotic concentrations in bone tissue that are challenging to achieve through systemic administration, while minimizing the side effects of long-term systemic therapy [7,8].

Antibiotic-loaded bone cement (ALBC) is based on polymethyl methacrylate (PMMA) and was introduced in the 1970s [9]. Nowadays, it is commercially available as ready-to-use pre-mixed formulations. ALBC delivers high local concentrations of antimicrobial agents directly to the site of implantation, for an extended period, thereby reducing the risk of infection while maintaining the mechanical stability of the implant [10,11]. These highly effective levels of antibiotics at the site are difficult to achieve with systemic administration, and local delivery minimizes the risk of systemic toxicity associated with high doses of antibiotics.

Gentamicin is a widely used antibiotic for local ODRI treatment due to its broad spectrum and concentration-dependent antibacterial activity [12]. Although gentamicin is often used as a single local therapy [13], in vitro studies have demonstrated that the combination of gentamicin plus clindamycin or vancomycin in bone cement can effectively reduce bacterial colonization and biofilm formation on the cement surface, suggesting potential clinical benefits in preventing and treating PJIs [10,12,14]. High-dose dual ALBC is especially beneficial as prophylaxis for patients with higher risk [11,15,16,17]. Continued research and clinical trials are necessary to fully understand the implications of using combined antibiotic therapies in bone cement for orthopedic infections. In vivo models play a major role in studying the pathogenesis of ODRIs, biofilm development in situ, and the efficacy of (novel) preventive or treatment strategies.

In translational research regarding ODRI, in vivo models are widely used to assess the biocompatibility and anti-microbial efficacy of antimicrobial coatings and compounds. Various animal models, including rodents (rats and mice), rabbits, dogs, and sheep, are used to study ODRI. Each model offers unique advantages depending on the study objectives, such as ease of handling, similarity to human bone structure, and the ability to monitor long-term outcomes. Although animal experiments are invaluable for advancing knowledge and developing interventions for ODRI, ethical concerns, translational limitations, high costs, variability, the complexity of models, and regulatory hurdles all pose challenges. Balancing these disadvantages with the potential benefits requires careful consideration, ongoing ethical review, and exploration of alternative research methods.

*Galleria mellonella*, commonly known as the greater wax moth, has emerged as a valuable model organism for studying microbial infections and the efficacy of antimicrobial agents [18]. Using *G. mellonella* simplifies ethical considerations, reduces costs, and speeds up preliminary research. Although there are limitations, such as the absence of adaptive immunity, *G. mellonella* provides critical insights into pathogenesis, host responses, and antimicrobial efficacy, paving the way for more detailed studies in mammalian models. Recently, we have developed a *G. mellonella* implant-associated infection model by either implanting *S. aureus* pre-incubated K-wires (i.e., an early-stage biofilm implant infection model) or implanting K-wires directly followed by an injection of *S. aureus* (i.e., a hematogenous implant infection model) and evaluated the efficiency of antibiotics and phages to prevent or treat implant infections [19,20].

Here, we aimed to utilize the *G. mellonella* larva implant infection model to evaluate the efficacy of commercially available ALBC against *S. aureus*. We successfully adapted the *Galleria mellonella* infection models to be used with antibiotic-loaded bone cements. This adaptation of the models allows further studies of the pathogenesis and prevention of PJIs in vivo.

## 2. Results

### 2.1. ALBC Discs Show Released Activity and Inhibit Bacterial Attachment In Vitro

**Figure 1:**
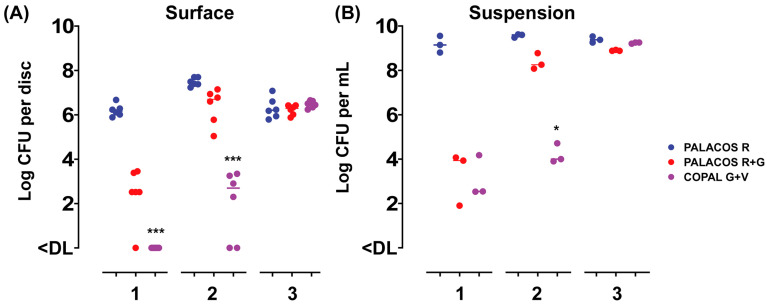
In vitro activity of the ALBC discs with (**A**) attachment to the disc surface and (**B**) growth in the suspension after 1–3 days of incubation, with a daily challenge of a fresh *S. aureus* EDCC 5055 inoculum suspension. Results are expressed as the numbers of viable bacteria retrieved from the disc surface (*n* = 6) and medium (*n* = 3). The horizontal lines represent the median values and are significantly different from the PALACOS R control groups as calculated by the Kruskal–Wallis rank sum test for the log CFU values (* = *p* < 0.05, *** = *p* < 0.001). The lower limit of detection (DL) is 25 and 5 CFU for the discs and medium, respectively.

To assess the release of the antibiotics from the ALBC discs over time, and the attachment of bacteria to the surface of the discs, the samples were incubated in a bacterial suspension for up to 3 days and challenged daily with a fresh inoculum suspension. After 1 day of incubation, PALACOS R+G showed a 3.7-log lower number of bacteria attached when compared to the control (PALACOS R; log 6.2 CFU/disc), whereas COPAL G+V (<DL; *p* < 0.001) fully prevented colonization by *S. aureus* on the surface of the discs (Figure 1A). The release of gentamicin alone (PALACOS R+G; log 3.9 CFU/mL) or in combination with vancomycin (COPAL G+V; log 2.5 CFU/mL) showed a 5.2- and 6.6-log lower number of bacteria in solution, respectively (Figure 1B). On the second day, the release of gentamicin alone (PALACOS R+G) resulted in only an approximately 1-log lower number of CFU on the surface (log 6.7 CFU/disc) and in the liquid (log 8.3 CFU/mL), when compared to the control (PALACOS R; log 7.4 CFU/disc and log 9.6 CFU/mL), and from the 3rd day on, there was no observable effect at all. Similarly, no effect was observed from day 3 onwards when gentamicin was combined with vancomycin (COPAL G+V); however, a larger reduction could be seen on day 2, with an approximately 5-log lower number of CFU on the surface (log 2.7 CFU/disc; *p* < 0.001) and in the liquid (log 4 CFU/mL; *p* < 0.05). Altogether, these results indicate that ALBC is able to prevent bacterial attachment and kill bacteria in the surroundings of the implants, with COPAL G+V being the most effective; however, in all cases, the effect decreases over time. This decreased activity over time is probably due to the dilution or even full removal of the released antimicrobial by the daily replacement of the medium.

### 2.2. ALBC Implants Prevent S. aureus Infections In Vivo

#### 2.2.1. ALBC Is Biocompatible in *G. mellonella* Larvae

**Figure 2:**
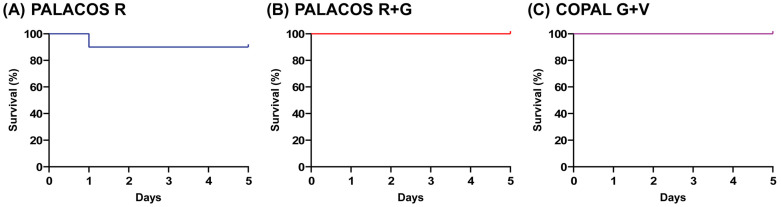
Biocompatibility assay. No significant differences are seen between the survival rates of larvae implanted with (**A**) the non-loaded control bone cement (PALACOS R), (**B**) ALBC with gentamicin alone (PALACOS R+G) or (**C**) ALBC with the combination of gentamicin and vancomycin (COPAL G+V). Experiments were conducted with 10 larvae per group.

The larvae receiving implants loaded with antibiotic (i.e., PALACOS R and COPAL G+V) were similarly active to the non-loaded PALACOS R group, and their survival rate at five days after implantation was 100% (PALACOS R+G and COPAL G+V) and 90% (PALACOS R), respectively (Figure 2). Thus, the implantation of ALBC did not cause any adverse effects such as wound healing disturbances, melanization at the site of implantation or toxicity due to the antibiotics released in case of PALACOS R+G and COPAL G+V.

#### 2.2.2. ALBC Implants Prevent Both Early-Stage Biofilm and Hematogenous Infections In Vivo

**Figure 3:**
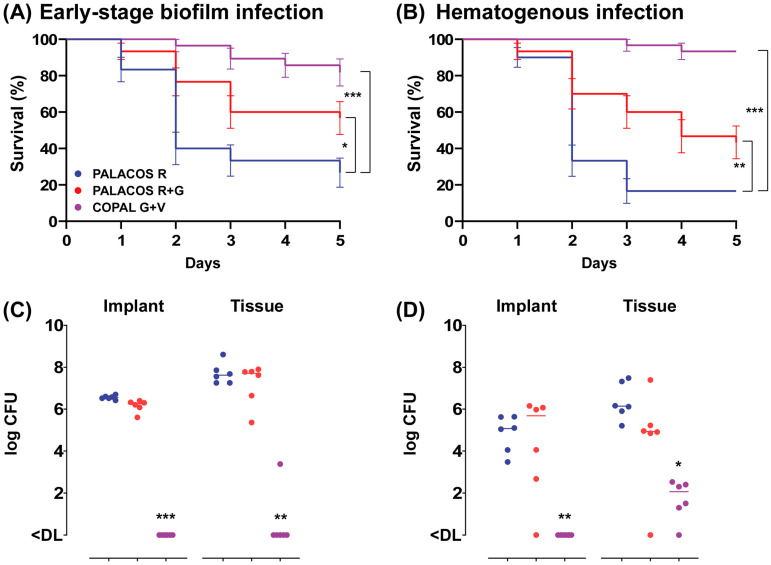
Prevention of (**A**) early-stage biofilm implant infection and (**B**) hematogenous implant infection by implantation of ALBC implants in *G. mellonella* larvae. For the early-stage biofilm infection, the ALBC implants were pre-incubated for 60 min in *S. aureus* solution (5 × 10^6^ CFU/mL) prior to implantation in the larvae. The mean number of bacteria before implantation was log 4.6 CFU (PALACOS R), log 3.4 CFU (PALACOS R+G) and log 2.4 CFU (COPAL G+V) per implant (*n* = 3 per group). In case of the hematogenous implant infection, the ALBC implants were first implanted in the larvae, followed by an injection with 10 µL of *S. aureus* inoculum (5 × 10^4^ CFU/larva) after 60 min. Percent survival (±SEM) over time (in days) is displayed after implantation of implants loaded with gentamicin alone (PALACOS R+G) or the combination of gentamicin and vancomycin (COPAL G+V), and non-loaded implants served as controls (PALACOS R). The data from three independent experiments were analyzed (*n* = 10 larvae per experiment) and statistical analysis was performed using a log rank test. The number of *S. aureus* on the ALBC implant (“Implant”) and in the tissue of the larvae (“Tissue”) after 24 h incubation in the (**C**) early-stage biofilm and (**D**) hematogenous implant infection model was determined. The horizontal lines represent the median values. Statistical analysis was performed using Kruskal–Wallis rank sum test (*n* = 6 per group). * = *p* < 0.05, ** = *p* < 0.01, *** = *p* < 0.001. The lower limit of detection (DL) is 5 and 3 CFU for the tissue and implants, respectively.

In order to assess the effectiveness of ALBC in preventing an early-stage *S. aureus* biofilm implant infection, we implanted ALBC implants, pre-incubated for 1 h in an *S. aureus* suspension, in the *G. mellonella* larvae. After 5 days of incubation at 37 °C, both types of ALBC implants significantly improved the survival of the larvae (Figure 3A, resulting in an improved survival rate with PALACOS R+G (57 ± 9%; *p* < 0.05) and COPAL G+V (82 ± 7%; *p* < 0.001), compared to the control (PALACOS R; 27 ± 8%).

To assess the ability of ALBC to prevent hematogenous implant infections, the larvae first received an implant and were infected with *S. aureus* 1 h later. The survival of the larvae was comparable to the early-stage biofilm model, with a moderate improvement in survival with PALACOS R+G (43 ± 9%; *p* < 0.01), but almost complete survival with COPAL G+V (93%; *p* < 0.001) compared to the non-loaded control (PALACOS R; 17%) (Figure 3B).

#### 2.2.3. ALBC Implants Eradicate Bacteria on Surface and in Tissue

At 1 day after implantation of the pre-incubated COPAL G+V implants, the bacteria were (almost) fully eradicated on the implant surface (<DL; *p* < 0.001) as well as in the larval tissue (<DL; *p* < 0.01), compared to the control group receiving PALACOS R (log 6.6 CFU/implant and log 7.6 CFU) (Figure 3C). However, PALACOS R+G hardly showed any reduction in numbers of CFU at all (log 6.3 CFU/implant and log 7.7 CFU in the tissue).

A similar effect is seen at 1 day after implantation and subsequent infection of the ALBC implants, mimicking hematogenous infections. COPAL G+V resulted in a significant 4.6-log and 4.1-log reduction in numbers of CFU on the implant surface (<DL; *p* < 0.01) and in the tissue (log 2.1 CFU; *p* < 0.05), respectively (Figure 3D). On the other hand, PALACOS R+G showed hardly any reduction in numbers of CFU (log 5.7 CFU/implant and log 4.9 CFU in the tissue), when compared to the control group receiving PALACOS R (log 5.1 CFU/implant and log 6.1 CFU in the tissue).

**Figure 4:**
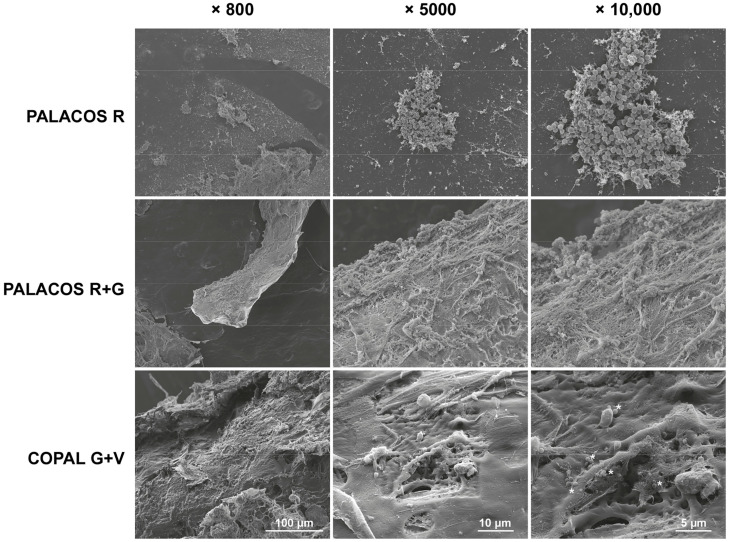
Scanning electron microscopy (SEM) analysis of *S. aureus* attachment on the different explanted ALBC implants after 24 h of incubation in the *G. mellonella* hematogenous implant infection model. The unloaded ALBC (PALACOS R) shows patches of bacteria, indicating possible biofilm formation, whereas the antibiotic-loaded implants show reduced (PALACOS R+G) or hardly any bacterial attachment (COPAL G+V); only individual bacteria could be observed (see *). Scale bars indicate 100 µm (800× magnification; left panels), 10 µm (5000× magnification; middle panels) or 5 µm (10,000× magnification; right panels).

These findings are confirmed by SEM analysis of explanted bone cement implants from the hematogenous infection model. A high number of bacteria colonized the surface of non-loaded ALBC implants (PALACOS R), with starting biofilm formation being observed, and less bacteria on the PALACOS R+G implants (Figure 4). In line with the quantitative culture results, only individual bacteria were seen on the surface of COPAL G+V.

## 3. Discussion

Commercially available (antibiotic-loaded) bone cements are widely used in clinics for the fixation of prostheses in primary total arthroplasties, fracture fixation or revision surgeries. In this study, we have adapted the *G. mellonella* early-stage biofilm and hematogenous implant infection models to be used with ALBC implants and subsequently evaluated the efficacy of these ALBCs against *S. aureus*. The overall outcome is the superior effect of the dual-loaded bone cement COPAL G+V in preventing infection compared to the limited effect of PALACOS R+G, which contains only gentamicin. In this study, we deliberately chose to use the clinically available formulations to be able to compare—or even validate—the outcomes of our models to the clinical practice. Thus, by using clinically relevant commercially available bone cements, we could mimic the clinical situation and thereby validate the model.

The elution of individual antibiotics from dual-loaded bone cement is better than when bone cement is loaded with a single antibiotic, thereby enhancing each other’s effect [21]. Dual ALBC is increasingly used in arthroplasty procedures after femoral neck fractures and demonstrates a reduction in PJI after hemiarthroplasty and seems, therefore, to be a useful method for the prevention of infection [17]. High-dose ALBC is especially beneficial for patients with increased risk factors for PJI [11,17], for instance, those with an intracapsular fracture of the hip [15]. However, in people aged 60 years or older receiving a hemiarthroplasty for intracapsular fracture of the hip, the use of high-dose dual-antibiotic loaded cement did not reduce the rate of deep surgical site infection in a randomized superiority trial [22].

Morris et al. used Dawley rats to establish a PJI knee model [23]. They implanted a porous titanium implant into the femur and an ultra-highly cross-linked polyethylene implant into the tibia, using gentamicin-coated bone cement (PALACOS R+G) to analyze the effectiveness against an *S. aureus* infection. Despite negative blood cultures with the gentamicin-loaded bone cement, *S. aureus* was still present in the joint tissue and on the implant surface. Similarly, our larvae model also revealed the presence of bacteria on both the surface and within the larvae tissue. Using a dual antibiotic-loaded (gentamicin and vancomycin) PMMA nail, rabbits with femoral osteomyelitis caused by a methicillin-resistant *Staphylococcus aureus* (MRSA) were successfully treated following surgical debridement and implantation [24]. Consistent with these findings, COPAL G+V achieved complete eradication of bacteria on the implant surface and a more than 2-log reduction in numbers of bacteria in the tissue in the *G. mellonella* implant infection model. The major advantages of using the *G. mellonella* implant infection model over other PJI models include the ability to conduct large cohort studies, high-throughput screening of new drugs, and testing various antibiotic combinations. This approach facilitates new drug development and reduces and refines the use of mammalian models in studies of orthopedic device-related infections [25].

Thus, we successfully adapted *G. mellonella* models for the use of ALBC. This is an important addition to the in vivo models, as bone cement is often used in the management of PJI for implant fixation or as a spacer. These spacers serve both therapeutic and mechanical functions during the interval between the removal of an infected prosthesis and the implantation of a new one. These in vivo models allow for detailed studies in the prevention or even treatment of PJI, in a relatively high-throughput, simple and cost-effective way. The ALBC released local high doses of antibiotics, without any signs of toxicity in the *G. mellonella* models. Unlike mammalian models, such as osteomyelitis mouse models [26,27], using *G. mellonella* raises fewer ethical concerns, making it easier to conduct large-scale studies without the need for extensive regulatory approval. Moreover, maintaining and handling *G. mellonella* is less expensive compared to vertebrate models, as they do not require specialized facilities, reducing overall research costs. The larvae are easy to handle and manipulate, and can be infected with a variety of pathogens, including bacteria and fungi. For example, we recently showed that *G. mellonella* can be used as an alternative in vivo models to study implant-associated fungal infections [28]. The life cycle of *G. mellonella* is relatively short and infection outcomes can be observed within days, allowing for quick assessment of pathogen virulence and treatment efficacy. They possess a complex innate immune system, including phagocytosis, melanization, and production of antimicrobial peptides, which provides insights into host-pathogen interactions relevant to higher organisms. On the other hand, unlike vertebrates, *G. mellonella* lacks an adaptive immune system and is unable to produce antibodies, which limits the ability to study long-term immune responses and vaccine efficacy. However, recent studies have demonstrated that insects possess mechanisms to maintain immunity, known as ‘immune priming’ [29]. Primary exposures of insects to bacteria and fungi lead to increased hemocyte production and enhanced resistance to subsequent infections by the same or similar pathogens [30,31]. Furthermore, Gallorini et al. established the immunophenotyping of hemocytes from infected *G. mellonella* larvae using cell membrane markers expressed by human immune cells [32]. This study highlights the analogies between vertebrate and invertebrate immune responses, as hemocytes react with anti-human antibodies. Consequently, this model could be used as a tool for screening new compounds, antibiotics, and vaccines. Lastly, results obtained from *G. mellonella* may not always translate directly to humans or other mammals due to differences in physiology and immune system complexity [18,33]. However, by using clinically applied ALBC in the current study, we could show a good correlation of the outcome in the in vivo model with the clinical practice.

In the future, the *G. mellonella* infection models could be used to study the pathogenesis of difficult-to-treat implant-associated infections, caused by multidrug-resistant (MDR) Gram-positive and Gram-negative bacterial strains belonging to the so-called ESKAPE panel (i.e., *Enterococcus faecium*, *S. aureus*, *Klebsiella pneumoniae*, *Acinetobacter baumannii*, *Pseudomonas aeruginosa*, and *Enterobacter* species) [34], also listed on the World Health Organization’s priority pathogen list [35,36]. The ESKAPE panel represents a global threat to human health because these bacterial strains can evade commonly used antibiotics. Therefore, the development of ALBC with broad-spectrum coverage needs to be considered in the fight against MDR bacteria. Moreover, these *G. mellonella* models could potentially be adapted to study infections of partially cemented metal implants, to further mimic clinical procedures. Therefore, *G. mellonella* implant infection models could be used to evaluate the effect of bone cement containing several combinations of antibiotics against multidrug-resistant pathogens.

## 4. Materials and Methods

### 4.1. Bacterial Cultures

The methicillin-sensitive *S. aureus* (MSSA) EDCC 5055, a strain with biofilm-forming capacity originally isolated from a wound infection, was used in the present study [37]. *S. aureus* EDCC 5055 is resistant to gentamicin (MIC: 4–8 mg/L; breakpoint: 2 mg/L) and susceptible to vancomycin (MIC: 1 mg/L; breakpoint: 2 mg/L), according to the EUCAST [38]. Prior to each experiment, bacteria from frozen stocks were grown overnight at 37 °C on LB agar plates (Carl Roth, Karlsruhe, Germany). From a single colony, an overnight culture was prepared in Brain-Heart Infusion (BHI; Merk, Darmstadt, Germany) broth by incubating at 37 °C and 180 rpm.

The overnight bacterial culture was diluted 100-fold in fresh BHI, and the bacteria were cultured to mid-logarithmic growth phase at 37 °C and 180 rpm, pelleted, washed once with phosphate-buffered saline (PBS; 140 mM NaCl, pH 7.4; Gibco, Life technologies, Paisley, UK), resuspended and diluted in BHI or PBS to 5 × 10^6^ CFU/mL for the in vivo experiments, based on the optical density of the suspension at 600 nm. The concentration of the inoculum suspension was verified by culturing duplicate 5 μL aliquots from 10-fold serial dilutions of the suspension on LB agar and determining the CFU/mL on the following day (quantitative culture).

### 4.2. Preparation of ALBC Discs and Implants

**Figure 5:**
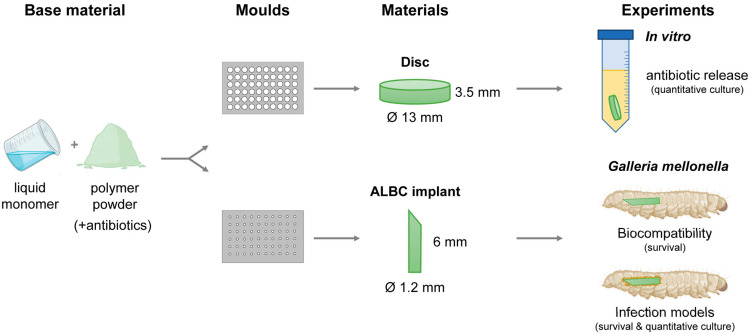
Development of experimental procedures. In short, the polymer powder was mixed with the monomer liquid and the resulting paste pressed into Teflon moulds to prepare uniform discs and cylindrical ALBC implants, for the in vitro and in vivo assays, respectively. The in vitro antimicrobial activity of the ALBC discs was determined. The in vivo biocompatibility was assessed in *G. mellonella*. Next, the in vivo effectivity was determined in the *G. mellonella* early-stage biofilm and hematogenous implant infection models.

The bone cements PALACOS R (containing no antibiotics; non-loaded), PALACOS R+G (containing 0.5 g gentamicin), and COPAL G+V (containing 0.5 g gentamicin and 2 g vancomycin) were obtained from Heraeus medical GmbH (Wehrheim, Germany). The radiopaque polymer (40–43 g powder, depending on the type of bone cement) was mixed well with 20 mL of monomer liquid in a bowl. The resulting paste was pressed into Teflon moulds (Karl Lettenbauer, Erlangen, Germany) using a spatula to prepare uniform discs (Ø 13 mm, 3.5 mm in height) and cylindrical implants (Ø 1.2 mm, 8 mm in length), for the in vitro and in vivo assays, respectively (Figure 5). After polymerization, the samples were removed from the moulds by applying force with a metal pin. Finally, the implants were sharpened using an electric combination tool (Georg Roth GmbH, Fürth, Germany).

### 4.3. In Vitro Antimicrobial Activity of ALBC Discs

Two ALBC discs were placed in an Erlenmeyer flask with 50 mL of an *S. aureus* inoculum suspension, prepared by diluting the overnight culture 1:100 in fresh BHI (containing ~1 × 10^7^ CFU/mL), and incubated at 37 °C and 100 rpm. After 24 h, the discs were rinsed with demineralized water, placed in fresh inoculum suspension and the process was repeated for up to 3 days. After incubation for 1–3 days, two measures of bacterial growth were quantified: the planktonic bacterial growth in the medium and the bacterial attachment and possible biofilm formation on the disc surface. Therefore, the medium was collected, and the discs were rinsed with demineralized water and then sonicated in 5 mL of PBS for 5 min at 45 kHz in a water bath sonicator (Ultrasonic Cleaner USC-T; VWR, Ismaning, Germany) and vortexed for 30 sec to detach and disperse adherent biofilm cells. This procedure does not affect bacterial viability [39]. The medium and sonicates were serially diluted tenfold and 5 droplets of 5 µL were plated on LB agar and incubated overnight at 37 °C. To increase the limit of detection, an additional 200 µL was plated on LB agar. The numbers of CFU per mL (medium) or per sample (discs) were determined after overnight incubation at 37 °C and expressed as log_10_ CFU per mL (medium) or as log_10_ CFU per sample (disc). The lower limit of detection was 5 CFU and 25 CFU for the medium and discs, respectively. For each group, 2 discs were incubated per Erlenmeyer flask, and each experiment was repeated 3 times. So, a total of *n* = 3 medium and *n* = 6 discs per group were used. To visualize the data on a logarithmic scale, a value of 1 CFU was assigned when no growth occurred.

### 4.4. G. mellonella Implant Infection Models

#### 4.4.1. Animals

*G. mellonella* larvae were ordered from Evergreen GmbH (Augsburg, Germany) and maintained on wheat germ (Tropic Shop GmbH, Nordhorn, Germany) at room temperature during the entire experiment. For each survival experiment, ten larvae in the last instar stage weighing around 500 mg were utilized per group, and each experiment was repeated 3 times (total of *n* = 30 larvae per group). To determine the number of bacteria on the implant surfaces and in the tissue of the larvae, 6 larvae per group were used.

#### 4.4.2. Early-Stage Biofilm Implant Infection Model

**Figure 6:**
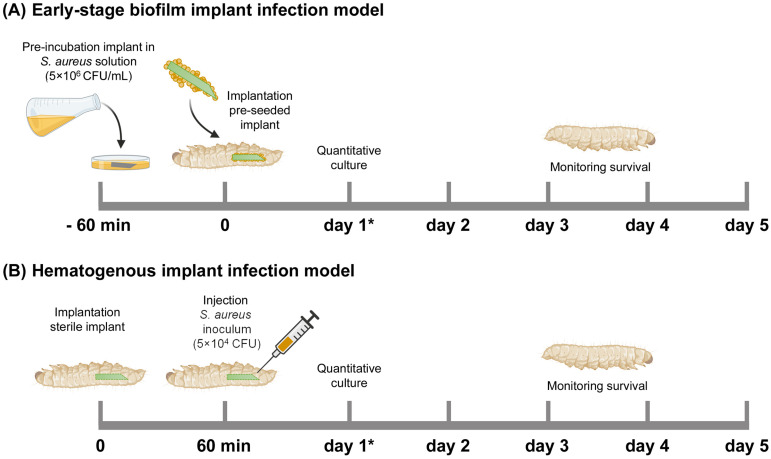
Schematic overview of the *Galleria mellonella* implant infection models used in this study. (**A**) Early-stage biofilm implant infection model: An ALBC implant was incubated in an *S. aureus* solution (5 × 10^6^ CFU/mL) for 60 min before implantation in the larva. (**B**) Hematogenous implant infection model: a sterile ALBC implant was implanted in the larva, and 10 µL *S. aureus* inoculum (5 × 10^4^ CFU/larva) was injected after 60 min. The survival of the larvae was monitored for 5 days. *N* = 30 larvae per experimental group. At 1 day after infection (see *), the number of CFU at the implant surface and in the tissue of the larvae was quantitatively determined (additional larvae, *n* = 6 per group).

For an early-stage biofilm infection, the cylindrical ALBC implants were pre-incubated in the *S. aureus* inoculum suspension in BHI (containing 5 × 10^6^ CFU/mL) for 1 h at 180 rpm, washed with PBS, and subsequently implanted at the rear end of the larvae by piercing their cuticle with the sharp end of the implant (Figure 6A). After implantation, the *G. mellonella* larvae were maintained at 37 °C and their survival was monitored for 5 days. Immediately before implantation, the number of bacteria on the surface of (additional; *n* = 3) the implants was determined according to the quantitative culture procedure in the following section.

#### 4.4.3. Hematogenous Implant Infection Model

To mimic the hematogenous infection route, a cylindrical ALBC implant was implanted in the larvae, as stated above, and incubated at 37 °C (Figure 6B). After 1 h, the larvae received an injection with 10 µL of an *S. aureus* inoculum suspension of 5 × 10^6^ CFU/mL in PBS (i.e., 5 × 10^4^ CFU/larva). The larvae were maintained and monitored as described above.

#### 4.4.4. Quantitative Culture

The antimicrobial effect of ALBC implants was determined by retrieving bacteria from the implant surface and from the tissue of the larva. At 1 day after implantation, the implants were separated from the tissue for the quantitative culture of bacteria. The implants were rinsed in demineralized water, sonicated in 0.5 mL PBS for 2 min at 45 kHz in a water bath sonicator and vortexed for 30 sec to dislodge all bacteria (Ultrasonic Cleaner USC-T; VWR, Ismaning, Germany). The tissue samples were homogenized in 1 mL of PBS using a combination of six large (Ø 2.8–3.2 mm) and ~15 smaller (Ø 1.4–1.6 mm) yttrium stabilized zirconium oxide grinding beads (Cerdur, Vechta, Germany) in the Precellys system (VWR), with six cycles of 30 s at 8000 rpm, with 30 s rest between cycles, under continuous cooling at 4 °C. The sonicates and homogenates were serially diluted tenfold and 5 droplets of 5 μL were plated on mannitol salt agar (MSA) plates (Sigma-Aldrich), to suppress growth of skin flora of the larvae, and incubated overnight at 37 °C. To increase the limit of detection, an additional 200 µL was plated. The numbers of CFU/sample were determined after overnight incubation at 37 °C and expressed as log_10_ CFU per implant or log_10_ CFU per larva. The lower limit of detection was 3 CFU and 5 CFU for implants and tissue, respectively. To visualize the data on a logarithmic scale, a value of 1 CFU was assigned when no growth occurred.

### 4.5. Scanning Electron Microscopy

Bacterial attachment to the ALBC implants, retrieved from the hematogenous implant infection model at 1 day, was studied using scanning electron microscopy (SEM). After removal from the larvae, the implants were washed twice with 1 mL of PBS to remove any non-adherent bacteria and fixed in 2.5% (*v*/*v*) glutaraldehyde (NeoFroxx GmbH, Einhausen, Germany) for 30 min at room temperature. Next, the implants were washed twice in PBS to remove the fixative and dehydrated in a graded ethanol concentration series (30%, 50%, 70%, 80% and 96%; Carl Roth) for 15 min each, followed by washing three times with 100% ethanol for 30 min. The implants were dried in a critical point dryer (EM CPD300, Leica, Wetzlar, Germany). Before imaging, samples were mounted on aluminum stubs (Ø 12.5 mm, Baltic Präparation, e.K., Wetter, Germany) and sputter-coated with a 4 nm gold–palladium layer (Polaron Sputter Coater SC760, Leica). Images were acquired at 15 kV using a LEO1530 (Ziess, Oberkochen, Germany). At least 10 fields of each implant were inspected and photographed at magnifications of 800×, 5000× and 10,000×.

### 4.6. Statistical Analysis

Statistical analysis of the data was performed using GraphPad Prism 9.5 (GraphPad Software, San Diego, CA, USA). For the analysis of bacterial numbers, the Kruskal–Wallis rank sum test, with Dunn’s correction for multiple comparisons, was applied, and the data were represented as log_10_ CFU with the median value per group. Differences between pairs of survival curves of the *G. mellonella* larvae were analyzed using the Mantel–Cox log rank test. The data were represented as means ± standard error of the mean from three independent experiments with 10 technical replicates of each survival experiment. The data were considered significant if the *p*-value was ≤0.05.

## 5. Conclusions

We successfully adapted *G. mellonella* implant infection models to be used with (antibiotic-loaded) cemented implants. First, bone cement, containing no antibiotics (PALACOS R), gentamicin alone (PALACOS R+G) or gentamicin in combination with vancomycin (COPAL G+V), was shaped into discs or cylindrical implants for the in vitro and in vivo experiments, respectively. Next, the cylindrical ALBC implants were implemented in the previously developed early-stage biofilm implant infection and hematogenous implant infection models [19]. To mimic an early-stage biofilm implant infection, the implants were incubated in an *S. aureus* solution for 60 min and subsequently implanted in the larvae. The hematogenous implant infection model was adapted as follows: a sterile ALBC implant was implanted in the larva, followed by an injection with *S. aureus* 60 min after implantation. In this way, both *G. mellonella* infection models can be used with ALBC to study the pathogenesis and prevention of PJIs in vivo. Thus, the *G. mellonella* larvae infection model with ALBC could be used as an alternative in vivo model to evaluate (novel) antimicrobial therapies against infections related to implant-related infections.

## Data Availability

The original contributions presented in the study are included in the article, further inquiries can be directed to the corresponding author.
